# Supplementary material for: MSC.sTRAIL Has Better Efficacy than MSC.FL-TRAIL and in Combination with AKTi Blocks Pro-Metastatic Cytokine Production in Prostate Cancer Cells
Source: Cancers (Basel). 2019 Apr 21;11(4):568. doi: 10.3390/cancers11040568 (PMC6521093; doi:10.3390/cancers11040568)
Supplement: Supplementary file 1 [file cancers-11-00568-s001.pdf]

Supplementary Material: MSC.sTRAIL Has Better Efficacy than MSC.FL-TRAIL and in Combination with AKTi Blocks Pro-Metastatic Cytokine Production in Prostate Cancer Cells

Andrea Mohr, Tianyuan Chu, Greg N. Brooke and Ralf M. Zwacka

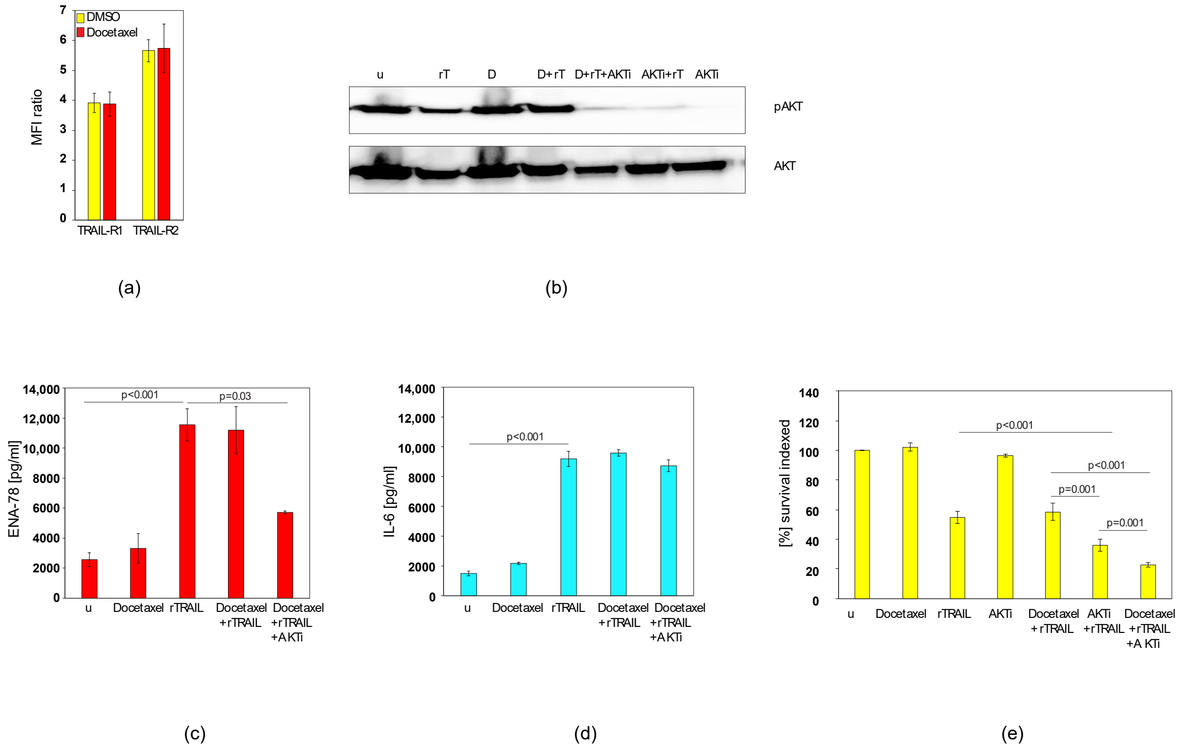

Figure S1. TRAIL-receptor expression, AKT phosphorylation and triple treatments. (**a**) Mean Fluorescence intensity ratios of TRAIL-R1 and TRAIL-2 on PC3 cells treated with docetaxel or with vehicle (DMSO) for 48 h. (**b**) Western blot of PC3 cell protein lysates that were treated with the indicated cocktails or left untreated (u) and probed with a phosphorylation specific AKT antibody and a complete AKT antibody as control. (**c**) CXCL5/ENA-78 ELISA results of supernatants of untreated PC3 cells (u), PC3 cells treated with docetaxel, treated with rTRAIL, treated with a mix of docetaxel and rTRAIL or a mix of docetaxel, rTRAIL and AKTi. (**d**) IL-6 ELISA results of supernatants of untreated PC3 cells (u), PC3 cells treated with docetaxel, treated with rTRAIL, treated with a mix of docetaxel and rTRAIL or a mix of docetaxel, rTRAIL and AKTi. (**e**) Survival of untreated PC3 cells (u), PC3 cells treated with docetaxel, treated with rTRAIL, treated with a mix of docetaxel and rTRAIL or a mix of docetaxel, rTRAIL and AKTi.

POS	POS	NEG	NEG	ENA-78 142.63	G-CSF 0.6	GM-CSF 42.48	GRO	GRO alpha	I-309	IL-1 alpha	IL-1 beta
IL-2	IL-3	IL-4	IL-5 1.95	IL-6 287.46	IL-7 31.66	IL-8	IL-10	IL-12	IL-13	IL-15	IFN-gamma
MCP-1	MCP-2	MCP-3	M-CSF	MDC	MIG 0.78	MIP-1	RANTES	SCF	SDF	TARC 2.03	TGF
TNF-alpha	TNF-beta	EGF	IGF	Angiogenin	OSM	TPO	VEGF-A	PDGF-BB	Leptin	NEG	POS

Figure S2. Cytokine Array indicating the order of cytokines on the array. Induced cytokines are boxed in red. Values depicted in these red boxes represent the upregulation in [%].

© 2019 by the authors. Licensee MDPI, Basel, Switzerland. This article is an open access article distributed under the terms and conditions of the Creative Commons Attribution (CC BY) license (http://creativecommons.org/licenses/by/4.0/).
